# Triggering Receptor Expressed on Myeloid Cells 2 Protects Dopaminergic Neurons by Promoting Autophagy in the Inflammatory Pathogenesis of Parkinson’s Disease

**DOI:** 10.3389/fnins.2021.745815

**Published:** 2021-11-18

**Authors:** Wei Huang, Qiankun Lv, Yunfei Xiao, Zhen Zhong, Binbin Hu, Si Yan, Yufang Yan, Junjun Zhang, Ting Shi, Lijuan Jiang, Wen Li, Guohui Lu

**Affiliations:** ^1^Department of Neurosurgery, The First Affiliated Hospital of Nanchang University, Nanchang, China; ^2^Department of Neurology, The Second Affiliated Hospital of Nanchang University, Nanchang, China; ^3^Institute of Translational Medicine, Nanchang University, Nanchang, China

**Keywords:** TREM2, inflammatory, autophagy, dopaminergic neurons, Parkinson’s disease

## Abstract

Parkinson’s disease is a neurodegenerative disorder with an inflammatory response as the core pathogenic mechanism. Previous human genetics findings support the view that the loss of TREM2 function will aggravate neurodegeneration, and TREM2 is one of the most highly expressed receptors in microglia. However, the role of TREM2 in the inflammatory mechanism of PD is not clear. In our study, it was found both *in vivo* and *in vitro* that the activation of microglia not only promoted the secretion of inflammatory factors but also decreased the level of TREM2 and inhibited the occurrence of autophagy. In contrast, an increase in the level of TREM2 decreased the expression of inflammatory factors and enhanced the level of autophagy through the p38 MAPK/mTOR pathway. Moreover, increased TREM2 expression significantly decreased the apoptosis of dopaminergic (DA) neurons and improved the motor ability of PD mice. In summary, TREM2 is an important link between the pathogenesis of PD and inflammation. Our study provides a new view for the mechanism of TREM2 in PD and reveals TREM2 as a potential therapeutic target for PD.

## Introduction

Parkinson’s disease is a neurodegeneration with the mechanism of central inflammation at its core (Subhramanyam et al., 2019; Pajares et al., 2020). In recent years, research on microglia has gradually become the focus of Parkinson’s disease research (Tang and Le, 2016). Microglia are a innate immune cell, and immune inflammation mediated by microglia should be associated with Parkinson’s disease (Kannarkat et al., 2013; Heneka et al., 2014). Activation of microglia was found both in autopsy of patients with PD and in MPTP-treated PD mice (Hirsch and Hunot, 2009; Yao et al., 2018). The activation of microglia leads to an increase in the levels of ROS and inflammatory factors, which in turn leads to the apoptosis of dopaminergic neurons, thus promoting the progression of PD (Anderson et al., 2018; Gan et al., 2019).

The Triggering Receptor Expressed on Myeloid Cells 2 (TREM2) gene, located on human chromosome 6 and mouse chromosome 17, encodes an innate immune receptor of the immunoglobulin family (Washington et al., 2002). In the brain, TREM2 is one of the most highly expressed receptors in microglia, and TREM2 is only expressed in microglia in the central nervous system (Yeh et al., 2017; McQuade and Blurton-Jones, 2019). It contains a long extracellular domain that regulates the function of microglia by stably interacting with DAP12 in the membrane and initiating the interaction between the signal transduction machinery and the extracellular environment (Konishi and Kiyama, 2018). The signal is terminated by TREM2 ectodomain shedding and subsequent intramembrane cleavage by γ-secretase (Steiner et al., 2020).

Triggering Receptor Expressed on Myeloid Cells 2 is associated with neurodegenerative diseases (Jay et al., 2017; Carmona et al., 2018; Ulland and Colonna, 2018). In Alzheimer’s disease (AD), TREM2 plays a protective role, and its overexpression is associated with the removal of soluble and insoluble Aβ42 aggregates from the brain (Lessard et al., 2018). Another report of Alzheimer’s disease is that the significantly decreased plaque load observed in APP transgenic and APOE or TREM2 knockout mice (Krasemann et al., 2017). At the same time, TREM2 is a potential genetic modifier of Huntington’s disease (HD), and its expression is related to the TLR4 receptor (Vuono et al., 2020). In vascular dementia (VD), TREM2 regulates the microglial activation phenotype to improve disease progression (Wang et al., 2020). In studies of these neurodegenerative diseases, which should be closely related to neuroinflammation, it has been demonstrated that TREM2 is involved in regulating the activation of microglia, which indicates that TREM2 improve disease progression through the regulation of neuroinflammation.

PD is a neurodegenerative disease with central chronic inflammation as the main pathogenic process (Rocha et al., 2018). At present, the role of TREM2 in this process is not clear. Therefore, we first determined the correlation between TREM2 and microglial activation in PD progression and showed that the expression of TREM2 could both inhibit the activation of microglia by inhibiting the p38 MAPK pathway and induce an increase in microglial autophagy by inhibiting the mTOR pathway, thereby reducing to reduce the pathological damage process of PD, protect dopaminergic neurons and improve motor symptoms of mice.

## Materials and Methods

### Cell Cultures and Treatment

We used murine microglial BV2 cells to examine the molecular mechanism of autophagy and the activation of microglia. BV2 cells were cultured in high-glucose DMEM (Solarbio, China) supplemented with 10% FBS (Gibco, United States) and 1% penicillin-streptomycin liquid (Solarbio, China). BV2 cells were cultured at 37°C in a humidified incubator with 95% air and 5% CO2. Lipopolysaccharide (LPS) was obtained from sigma (L2630, United States). 3-MA was obtained from sigma (189490, United States).

### Transfection

The pEGFP-C1-Trem2-m (P9057) plasmid and the pEGFP-C1 (P0314) were obtained from Biolink (Miaoling, China). Plasmids were transfected using the Lipofectamine 3000 kit (Invitrogen, Carlsbad, CA, United States).

### Animals and Treatment

Six- to eight-week-old male C57BL/6 mice were purchased from Hunan Sja Laboratory Animal Co., Ltd. Male mice were selected to rule out the neuroprotective activities of estrogens, progesterone and certain other neuroactive steroids on mesencephalic DA neurons (Isenbrandt et al., 2021). The animals were fed in a controlled environment and provided standard rodent chow and water. The implementation of animal care and procedures was approved by the Ethics Committee of the Second Affiliated Hospital of Nanchang University and complied with the guidelines for laboratory animal care and use from the National Institutes of Health (NIH). The mice were divided into the following four groups: saline, 0, 7, and 21 days. Then, the mice in the 0, 7, and 21 days groups received 1 i.p. injection of MPTP-HCl (20 mg/kg free base, M0896, Sigma, United States) per day for five consecutive days (Yao et al., 2019). The mice in the saline group received 1 i.p. injection of saline per day for 5 days. The mice were decapitated at specific times, their brains were removed, and midbrain samples including the substantia nigra pars compacta (SNpc) were stored at −80°C.

### Lentiviral Vector Construction and Stereotaxic Surgery

The lentivirus vectors encoding the mouse TREM2 gene (NCBI ID: NM_031254.3) into the multicloning site of the lentivirus backbone plasmid of pHBLV-CMV-Zsgreen-puro. The constructs were cotransfected with packaging vectors into 293T cells for packaging followed by purification (Hanbio Co. LTD., Shanghai, China). Stereotaxic surgery was performed with a stereotaxic frame (Stoelting, Wood Dale, IL, United States) and a 5 μl Hamilton syringe fitted with a pulled glass capillary tube. After anesthesia, the head of the mouse was fixed in the flat-skull position for stereotactic operation. Lentiviruses (3 μl; 1 × 10^10^ IFU/μl per construct) were stereotaxically delivered into the right substantia nigra region (AP: 3.1 mm, ML: 1.2 mm, DV: 5.1 mm from bregma) as previously reported (Ye et al., 2018). One week later, the mice were treated with saline or MPTP according to the above methods. All mice were killed at a specified time, and the midbrain was stored at −80°C.

### RNA Extraction and Quantitative RT–PCR Analysis

Total RNA was extracted from cells and mouse brain tissue samples using TriQuick Reagent (Solarbio, China). A NanoDrop ND-2000 Spectrophotometer (Thermo Fisher Scientific, Inc., Wilmington, DE, United States) was used to quantify the RNA concentration. For assessments of mRNA expression, 1 μg of total RNA was used to synthesize the complementary DNA using the PrimeScript^TM^ RT Reagent Kit with gDNA Eraser (Takara, Japan). cDNA products were then diluted 1:10 in ddH2O. RT-qPCR was performed using the FastFire qPCR PreMix (Tiangen, China). The primer sequences were as follows: TREM2 (forward): 5′-CG GGATCCATGGGACCTCTCCACCAGTTTC-3′, (reverse) 5′-CCGCTCGAGAGCAAAAGTAGCAGAAACAGA-3′; GAPDH (forward): 5′-GGGAAATTCAACGGCACAGT-3′, (reverse) 5′-AGATGGTGATGGGCTTCCC-3′; TNF-a (Forward): 5′-TATG GCTCAGGGTCCAACTC-3′, (reverse) 5′- GGAAAGCCCA TTTGAGTCCT-3′; IL-1β (forward): 5′-CTCACAAGCAGA GCACAAGC-3′, (reverse) 5′-CAGTCCAGCCCATACTTT AGG-3′. The expression level of each sample was compared with the expression of GAPDH as an internal control. Data are from three separate experiments, and each experiment was performed in triplicate.

### Western Blot Analysis

The protein levels of TREM2, LC3, p38, p-p38, mTOR, and p-mTOR in cultured cells and mouse brain tissue samples were detected by Western blotting. Total protein was extracted using RIPA lysis buffer (P0013B; Beyotime, China) containing protease and phosphatase inhibitors (P1082; Beyotime, China). Protein concentration was measured by bicinchoninic acid protein assay (Thermo Fisher Scientific, Inc., United States). Equal amounts of protein were separated using SDS-PAGE (BOSTER, China) and then transferred to a PVDF membrane (Solarbio, China). Membranes were then blocked with 5% non-fat dry milk in TBS with 0.1% Tween and probed with the appropriate primary antibodies against TREM2 (1:1,000, ABclonal, A10482), LC3B (1:1000, Cell Signaling, #2775), p-p38 (1:1000, Cell Signaling, #9216), p38 (1:1000, Cell Signaling, #9212), p-mTOR (1:1000, Cell Signaling, #2971), mTOR (Cell Signaling, #2972), GAPDH (1:1000, Servicebio, GB12002), and β-actin (Cell Signaling, #3700) overnight at 4°C. HRP-conjugated secondary antibodies (goat anti-mouse/rabbit IgG, 1:5000) were used for antibody detection.

### Cytokine Assays

BV2 cells (1 × 10^5^) were seeded onto 24-well plates and incubated overnight. Cells were transfected with TREM2 plasmid or NC plasmid for 24 h and then stimulated by LPS (1000 ng/ml) for 24 h. The concentrations of proinflammatory cytokines TNF-a and IL-1β in culture supernatants were determined by ELISA kits (PT512, PI301, Beyotime, China) according to the manufacturer’s protocol.

### Behavioral Tests

The rotarod test and pole-climbing test were used to evaluate the coordination and balance of mouse movement as previously reported (Han et al., 2020). In the rotarod test, the mice were first trained on the instrument at a constant speed of 20 rpm for 5 min. Then, the mice were placed on the accelerator rod (4 to 40 rpm) for 300 s, and the time the mice stayed on the accelerator rod (the latency of decline) was recorded. In the pole-climbing test, a thick wooden pole with a diameter of 1 cm and a length of 50 cm and a thick wooden ball with a diameter of 2 cm at the top were fixed vertically on the base. The mice were placed on the ball and allowed to climb down. The time from the release of the ball to the mouse forelimb reaching the bottom of the club was recorded. Each mouse carried out three consecutive experiments with intervals of 30 min, and the average time was recorded in the process of data processing.

### Immunohistochemistry and Immunofluorescence

Animals were anesthetized with sodium pentobarbital at 7 days after MPTP administration. Brains were removed, post-fixed in 4% paraformaldehyde for 24 h, and cryopreserved in 30% sucrose for 48 h. The frozen brains were then coronally sectioned at a thickness of 20 μm on a cryomicrotome, and the sections were mounted on slides. Dopaminergic neurons were identified using a primary antibody against TH (25859-1-AP, Proteintech) and a biotinylated secondary antibody and streptavidin ABC solution. Immunostaining images were obtained with an Aperio GT450 (Leica, Germany). For immunofluorescence staining, sections were first incubated at 4°C overnight with the following antibodies: mouse anti-Iba1 (Servicebio, GB12105),rabbit anti -NeuN (Servicebio, GB11138). This was followed by incubation with Alexa Fluor-488 or Alexa Fluor-546-conjugated secondary antibody at room temperature for 2 h. Immunofluorescent staining images were obtained with a Pannoramic MIDI (3DHISTECH, Hungary).

### Statistical Analysis

All data are expressed as the mean ± standard error of the mean (SEM). SPSS version 19 (SPSS, Inc., Chicago, IL, United States) was used for statistical analysis. A double-tailed unpaired *t*-test was used for comparisons between the two groups, and the difference was statistically significant. Single factor analysis of variance (ANOVA) was used for comparison among groups, and the Bonferroni post-test was used. When *P* < 0.05, the difference was statistically significant.

## Results

### The Expression Level of Triggering Receptor Expressed on Myeloid Cells 2 Is Downregulated in Lipopolysaccharide-Induced BV2 Cells

**FIGURE 1:**
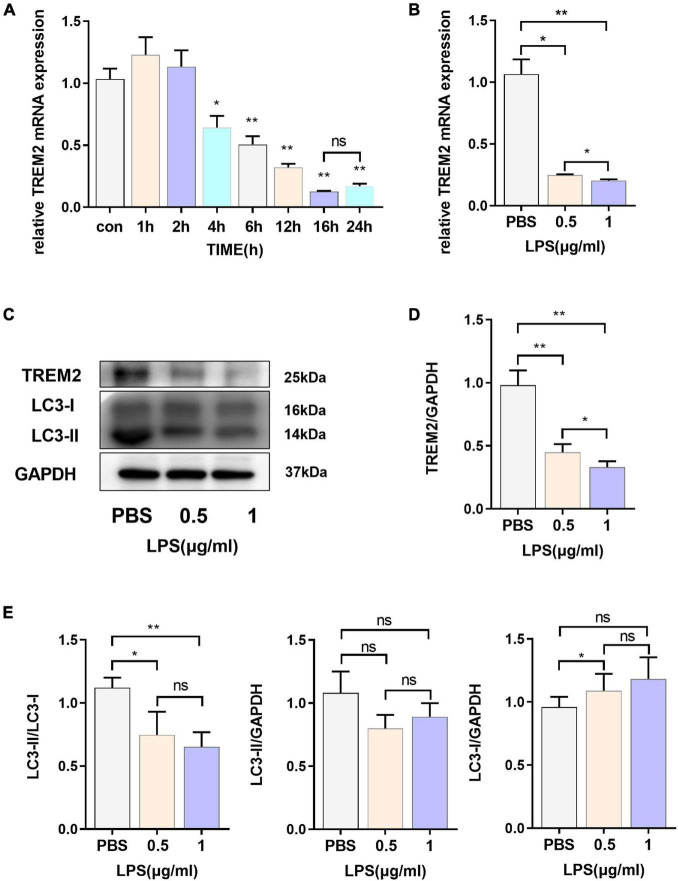
The express ion levels of TREM2 are down-regulated in LPS-induced BV2 cells. **(A)** After BV2 cells were incubated with 1 μg/ml LPS for different times (0, 1, 2, 4, 6, 12, 16, and 24 h), the mRNA expression level of TREM2 was detected by RT-qPCR (*n* = 3/group) (*F* = 77.91). **(B)** After BV2 cells were incubated with 0.5 or 1 μg/ml LPS for 24 h, the mRNA expression level of TREM2 was detected by RT-qPCR (*n* = 3/group) (*F* = 148.3). **(C)** After cells were treated with different concentrations of LPS (0.5 or 1 μg/ml) for 24 h, the expression of TREM2 and LC3 were detected by WB (*n* = 3/group). **(D)** The expression level of TREM2 was analyzed by quantitative analysis of protein (*F* = 53.4). **(E)** The expression level of LC3II and LC3I was analyzed by quantitative analysis of protein, and the ratio of LC3II/LC3I was shown (*F* = 15.93). All the quantitative analysis of protein was done by Image J to calculate the gray value. Data are shown as the mean ± SE. The fold change is significant where *P < 0.05, **P < 0.01. *n* = number of independent cell culture preparations.

In our study, we evaluated the expression of TREM2 in BV2 cell lines induced by lipopolysaccharide (LPS) and found that TREM2 expression increased briefly after the cells were stimulated by LPS for a short period of time, and the expression level of TREM2 mRNA decreased with time (Figure 1A) (*p* < 0.05). According to our results, the change in TREM2 expression level was more striking after 16-h treatment. We treated the cells with LPS (0.5 or 1 μg/ml) for 24 h and then detected the expression levels of TREM2 and LC3 protein (Figure 1C). The results showed that the TREM2 protein level decreased significantly under LPS treatment (Figure 1D) (*p* < 0.01), and so did the expression of mRNA (Figure 1B) (*p* < 0.05). Moreover, pretreatment with LPS (0.5 or 1 μg/ml) for 24 h suppressed autophagy as determined by the ratio of LC3II/I (Figure 1E) (*P* < 0.05).

### Overexpression of Triggering Receptor Expressed on Myeloid Cells 2 Could Effectively Promote Microglial Autophagy

**FIGURE 2:**
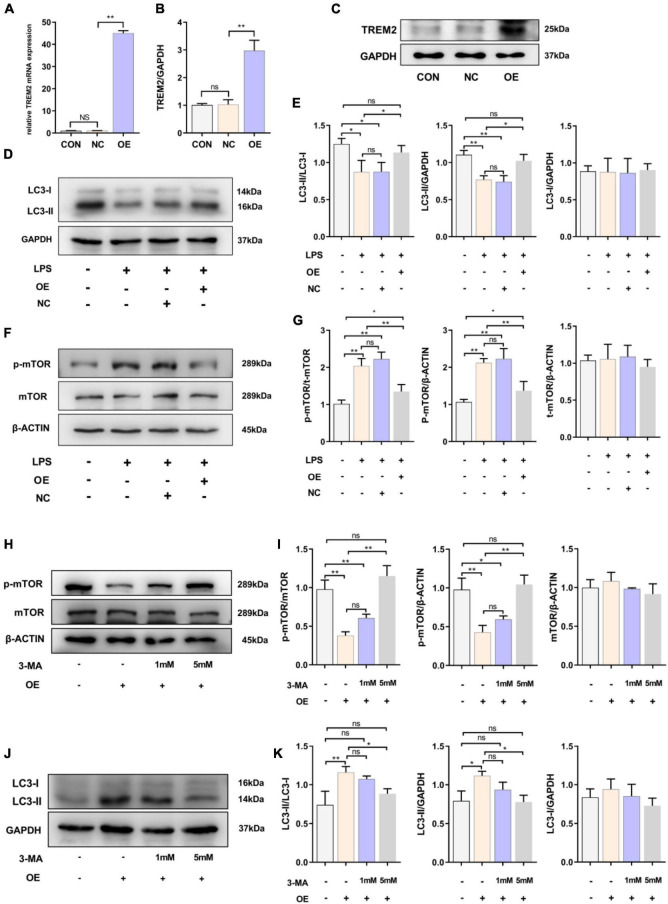
Overexpression of TREM2 significantly increased the autophagy level and suppressed the p-mTOR level of BV2 cells treated with LPS. **(A)** After BV2 cells were transfected with plasmid 24 h, the mRNA expression level of TREM2 was detected by qPCR (*n* = 3/group). **(B,C)** The expression level of TREM2 protein after transfection of plasmid was detected by WB (*n* = 3/group), and the expression level of TREM2 protein was analyzed by quantitative analysis of protein (*F* = 30.38). **(D,E)** The expression level of LC3 protein after transfection of plasmid was detected by WB (*n* = 3/group), and the expression of LC3II and LC3I was analyzed by quantitative analysis of protein, the ratio of LC3II/LC3I was shown (*F* = 8.04). **(F,G)** The expression level of p-mTOR and mTOR protein after transfection of plasmid was detected by WB (*n* = 3/group), and the expression of p-mTOR and mTOR were analyzed by quantitative analysis of protein, the ratio of p-mTOR/mTOR was shown (*F* = 31.84). **(H)** After the cells were transfected with plasmids, the cells were treated with 1 or 5 mM 3-MA for 24 h, the expression level of p-mTOR and mTOR protein was detected by WB. **(I)** The ratio of p-mTOR/mTOR was shown (*F* = 39.48). **(J)** After the cells were transfected with plasmids and treated with 3-MA, the LC3 was detected by WB (*n* = 3/group). **(K)** The ratio of LC3II/LC3I was shown (*F* = 10.19). NC stands for transfection of pEGFP-C1 (empty-plasmid). OE stands for transfection of TREM2-plasmid. All the quantitative analysis of protein was done by Image J to calculate the gray value. Data are shown as the mean ± SE. The fold change is significant where *P < 0.05, **P < 0.01. *n* = number of independent cell culture preparations.

Because LPS stimulated the expression of TREM2, we speculated that it could play a role in the activation of the BV2 cell line. After 24 h of LPS stimulation, the level of LC3-II in BV2 cells decreased, and autophagy was inhibited. Therefore, we then overexpressed TREM2 in the BV2 cell line and further verify the role of TREM2 overexpression in microglia. We verified the expression of TREM2 after TREM2 plasmid or control plasmid transfection by RT-qPCR (Figure 2A) (*p* < 0.01). In addition, we evaluated the TREM2 protein level by WB (Figures 2B,C) (*p* < 0.01). Compared to the NC group (transfected control plasmid), the cells transfected with TREM2 plasmid showed a higher ratio of LC3II/I following a 24 h incubation with LPS (Figures 2D,E) (all *P* < 0.05). Earlier studies have shown that autophagy is mainly regulated by the mTOR pathway. It was previously shown that LPS stimulation can activate the mTOR pathway (Sun et al., 2017). We have shown that TREM2 can enhance microglial autophagy induced by LPS, but it is not clear whether this process is carried out through the mTOR pathway. Then we evaluated the expression level of p-mTOR under LPS treatment after we overexpressed the TREM2. We found that, compared with the NC group, overexpressed the TREM2 inhibited the expression of p-mTOR and reduced the ratio of p-mTOR/mTOR induced by LPS (Figures 2F,G) (*P* < 0.01). To further elucidate the mechanism of TREM2-induced autophagy, we transfected TREM2 overexpression plasmid into BV2 cells and then we treated BV2 cells with 1 or 5 mM 3-MA for 24 h to inhibit autophagy. Overexpression of TREM2 could inhibit the expression of p-mTOR and promote autophagy, while 3-MA can inhibit this process (Figures 2H–K). This suggested that TREM2 regulate autophagy through mTOR pathway.

### Overexpression of Triggering Receptor Expressed on Myeloid Cells 2 Could Effectively Attenuate Lipopolysaccharide-Induced BV2 Microglial Activation

**FIGURE 3:**
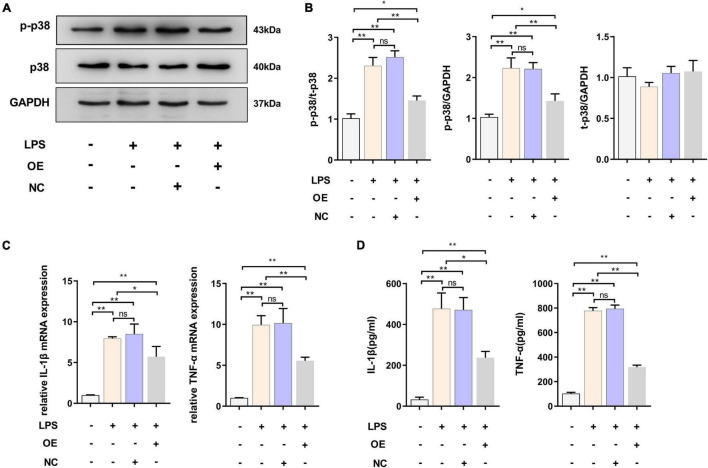
Overexpressed TREM2 inhibits the P38 MAPK pathway and proinflammatory cytokines. **(A,B)** BV2 cells were transfected with plasmid 24 h, then the cells were incubated with LPS (1 μg/ml) for 24 h. The expression level of p-p38 and p38 protein after transfection of plasmid was detected by WB (*n* = 3/group), and the expression of p-p38 and p38 were analyzed by quantitative analysis of protein, the ratio of p-p38/p38 was shown (*F* = 68.49). **(C)** The mRNA levels of the proinflammatory cytokines TNF-α (*F* = 59.38) and IL-1β (*F* = 45.76) were determined using RT-qPCR (*n* = 3/group). **(D)** The protein levels of the proinflammatory cytokines TNF-α (*F* = 78.72) and IL-1β (*F* = 51.53) were determined using ELISA (*n* = 3/group). NC stands for transfection of empty-plasmid. TREM2 OE stands for transfection of TREM2-plasmid. All the quantitative analysis of protein was done by Image J to calculate the gray value. Data are shown as the mean ± SE. The fold change is significant where *P < 0.05, **P < 0.01. *n* = number of independent cell culture preparations.

We demonstrated that the expression level of TREM2 decreased in the activated BV2 cells. The downregulation of TREM2 in response to LPS stimulation suggested that TREM2 might be involved in the regulation of the microglial response to LPS. We then determined whether the over-expression of TREM2 could attenuate LPS-induced BV2 microglial activation. To assess this issue, the over-expression of TREM2 in microglia was examined. We transfected TREM2 overexpression plasmid into BV2 cells and evaluated the expression level of p-p38 under LPS treatment (Figure 3A). Compared to the NC group, the cells transfected with TREM2 overexpression plasmid showed a lower ratio of p-p38/p38 following a 24 h incubation with LPS (Figure 3B) (*P* < 0.05). Over-activated microglia can mediate the detrimental effects of neurotoxicity and inflammation through the excess production of cytotoxic factors (Leng and Edison, 2020). We then investigated the expression of TNF-α and IL-1β mRNA and protein levels by RT-qPCR and ELISA following a 24 h incubation with LPS. We found that the expression of LPS-induced TNF-α and IL-1β in the TREM2 OE group was significantly lower than that in the NC group (Figures 3C,D) (*P* < 0.05). Taken together, the over-expression of TREM2 could effectively suppress LPS-induced microglial activation.

### Expression Levels of Triggering Receptor Expressed on Myeloid Cells 2 and Activated Microglia in the Substantia Nigra Pars Compacta of MPTP-Treated Mice *in vivo*

**FIGURE 4:**
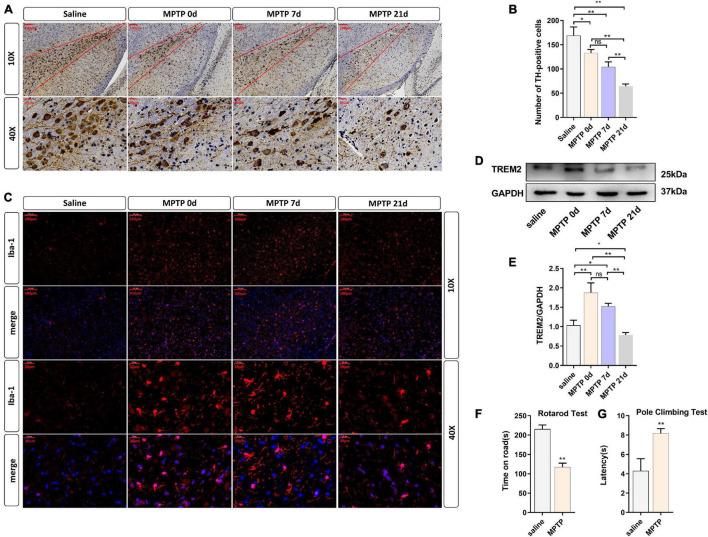
TREM2 expression and microglial activity were observed in the SNpc of mice treated with MPTP. The mice were intraperitoneally injected with MPTP-HCI once a day for 5 days, and the control group was injected with normal saline. Then, the mice were sacrificed by decapitation at different time points (0, 7, or 21 days) after the last injection of MPTP, and the midbrain tissue was collected. 0 day (immediately after the last MPTP injection). **(A)** Immunostaining of tyrosine hydroxylase (TH)-positive neurons in the SNPC is shown (*n* = 3/group). Scale bar: 100 μm and 20 μm. **(B)** The number of TH cells in the SNpc (the area between two red lines) were manually counted (*F* = 48.73). **(C)** Immunofluorescence images confirmed the expression of Iba-1 (*n* = 3/group). Red: anti-Iba1 (antibody labeling microglia). Scale bar: 100 μm and 20 μm. **(D)** After MPTP injection, the expression level of TREM2 protein was detected by WB (*n* = 3/group). **(E)** The expression level of TREM2 protein was analyzed by quantitative analysis of protein (*F* = 35.52). All the quantitative analysis of protein was done by Image J to calculate the gray value. **(F,G)** The pole-climbing test and rotarod test for bradykinesia were performed on 7th day after the last MPTP injection (*n* = 12/group). Data are shown as the mean ± SE. The fold change is significant where *P < 0.05, **P < 0.01. *n* = number of animals.

To further verify the role of TREM2 in Parkinson’s disease, we constructed an MPTP PD model. In this experiment, mice were injected with saline or MPTP at different times, and the changes in TREM2 expression were measured. First, we confirmed by immunohistochemistry the damage to dopaminergic neurons induced by MPTP (Figure 4A). Next, we manually count the number of TH-positive cells in the SNpc (the area between two red lines) (Figure 4B). Then, we observed by immunofluorescence that the activation level of microglia in the mouse brain increased after the MPTP injection (Figure 4C). In order to verify whether TREM2 changes in the brain of mouse induced by MPTP, we detected the expression level of TREM2 by WB. We found that the expression level of TREM2 increased on the 7th day and then returned to normal on the 21st day (Figures 4D,E), which is consistent with previous reports (Zhang et al., 2018). At the same time, we verified the damage of MPTP on the motor ability of mice through behavioral experiments, the results of pole-climbing test and rotarod test for bradykinesia showed that MPTP impaired the motor ability of mice (Figures 4F,G). We speculate that the expression of TREM2 could antagonize the activation of microglia.

### Overexpressed Triggering Receptor Expressed on Myeloid Cells 2 Inhibited the Activation of Microglial Cells, Inhibited the Apoptosis of Dopaminergic Neurons, and Improved the Motor Ability of MPTP-Treated Mice

**FIGURE 5:**
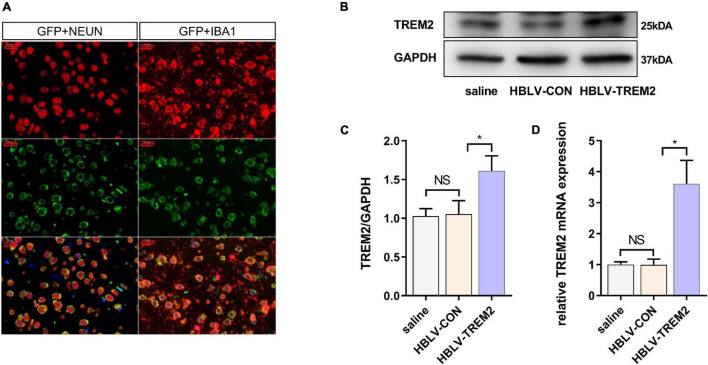
HBLV-TREM2 was transferred into neurons and microglia, promoted the expression of TREM2 in SNpc. The mice were subjected to stereotactic intraventricular treatment with HBLV-TREM2. Then, the mice were decapitated, and the midbrain was obtained 7 days after the HBLV-TREM2 injection. **(A)** Immunofluorescence of GFP marker and Iba1 or Neun are shown (*n* = 1/group). Red: anti-Iba1 (antibody labeling microglia) and NeuN (antibody labeling neurons). Green: GFP marker. Scale bar: 100 μm and 20 μm. **(B)** Seven days after injection of the HBLV-TREM2 or HBLV-NC, the TREM2 protein was detected by WB (*n* = 3/group). **(C)** The expression level of TREM2 protein was analyzed by quantitative analysis of protein (*F* = 12.66). **(D)** The mRNA expression level of TREM2 was detected by qPCR (*n* = 3/group) (*F* = 33.59). All the quantitative analysis of protein was done by Image J to calculate the gray value. Data are shown as the mean ± SE. The fold change is significant where *P < 0.05, **P < 0.01. *n* = number of animals.

**FIGURE 6:**
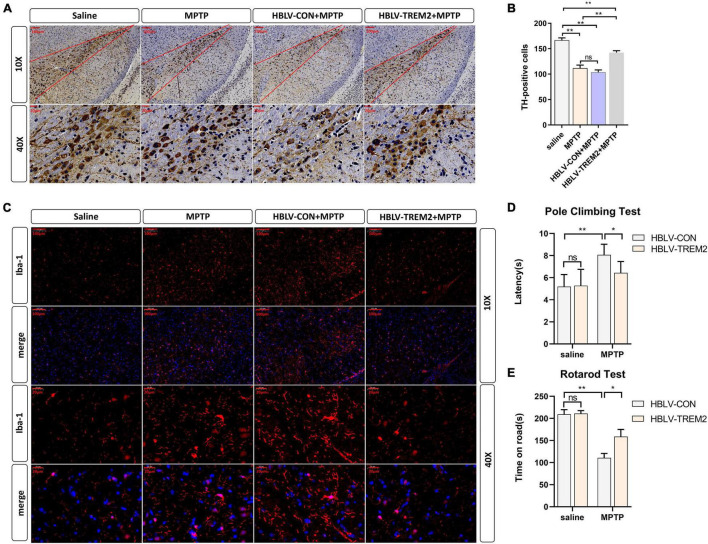
Overexpression of TREM2 can inhibit the activation of microglia, reduce the damage to DA neurons, and improve the motor ability of MPTP-treated mice. The mice were subjected to stereotactic intraventricular treatment with HBLV-TREM2 or NC and then received 1 intraperitoneal injection of MPTP-HCl per day for 5 days. The HBLV-TREM2 treatments were performed 7 days prior to the MPTP injection. At the same time, mice that were not injected with the HBLV-TREM2 or NC received MPTP-HCl injections, and the control mice received saline injections. Then, the mice were decapitated, and the midbrain was obtained 7 days after the last MPTP injection. **(A)** Immunostaining of tyrosine hydroxylase (TH)-positive neurons in the SNPC is shown (*n* = 3/group). Scale bar: 100 μm and 20 μm. **(B)** The number of TH cells in the SNpc (the area between two red lines) were manually counted (*F* = 109.2). **(C)** Immunofluorescence images confirmed the expression of Iba-1 (*n* = 3/group). Red: anti-Iba1 (antibody labeling microglia). Scale bar: 100 μm and 20 μm. **(D,E)** The pole-climbing test and rotarod test for bradykinesia were performed on 7th day after the last MPTP injection (*n* = 12/group). All the quantitative analysis of protein was done by Image J to calculate the gray value. Data are shown as the mean ± SE. The fold change is significant where *P < 0.05, **P < 0.01. *n* = number of animals.

Given that TREM2 regulates the expression of p-p38/p-mTOR, which is involved in the pathogenesis of inflammation, to inhibit inflammation *in vitro*, we next studied whether TREM2 can inhibit the activation of microglia in a PD mouse model. As reported in previous studies, we injected HBLV-TREM2 or HBLV-NC into the right SNpc (AP: 3.1 mm, ML: 1.2 mm, DV: 5.1 mm from bregma) (Ye et al., 2018). One week after the lentivirus injection, we detected the infection efficiency of the lentivirus by observing the fluorescence of the lentiviral GFP marker, we found that the lentivirus has different degrees of infection to microglia and neurons in SNpc of brain (Figure 5A). Concurrently, we examined the mRNA and protein expression of TREM2 in SNpc by RT-qPCR and WB (Figures 5B–D). One week after the lentivirus injection, we began to inject MPTP into the mice. Then, on the 7th day after the completion of the MPTP injection, we conducted a follow-up experiment. Through the immunohistochemical experiment, by manually counting the number of TH-positive cells in the SNpc, we found that the dopaminergic neurons of MPTP mice injected with TREM2 lentivirus were protected compared with those of the NC group (Figures 6A,B). Next, we observed by immunofluorescence that the activation level of microglia in the mouse brain (Figure 6C). Finally, we verified the protection of TREM2 on the motor ability of PD mice through behavioral experiments, the motor ability of MPTP-treated mice injected with HBLV-TREM2 was improved compared with the NC group (Figures 6D,E).

### Overexpressed Triggering Receptor Expressed on Myeloid Cells 2 Inhibits the Expression of p-p38, p-mTOR, Promote Autophagy in Substantia Nigra Pars Compacta of MPTP-Treated Mice

**FIGURE 7:**
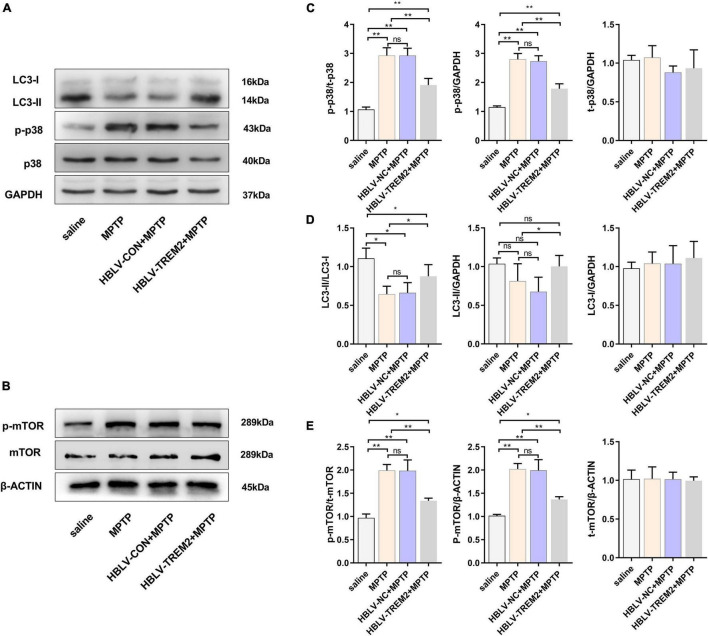
Overexpression of TREM2 can inhibit the expression of p-p38, p-mTOR, and promote autophagy in SNpc of MPTP-treated mice. After the mice were injected with lentivirus and MPTP, the mice were decapitated, and the midbrain was obtained 7 days after the last MPTP injection. **(A,B)** The expression of TREM2, LC3, p-p38, p38, p-mTOR, mTOR were detected by WB (*n* = 3/group). **(C)** The expression of p-p38 and p38 were analyzed by quantitative analysis of protein, the ratio of p-p38/p38 was shown (*F* = 50.30). **(D)** The expression of LC3 was analyzed by quantitative analysis of protein, the ratio of LC3II/LC3I was shown (*F* = 8.36). **(E)** The expression of p-mTOR and mTOR were analyzed by quantitative analysis of protein, the ratio of p-mTOR/mTOR was shown (*F* = 38.15). All the quantitative analysis of protein was done by Image J to calculate the gray value. Data are shown as the mean ± SE. The fold change is significant where *P < 0.05, **P < 0.01. *n* = number of animals.

To further verify the mechanism of TREM2 in the PD model, we observed the expression level of LC3, p-p38, p38, p-mTOR, mTOR by WB (Figures 7A,B). Studies have shown that MPTP can promote the expression of p-p38, while TREM2 can inhibit this process (Figure 7C). Compared with the NC group, TREM2 inhibited the expression of p-mTOR, activated autophagy and promoted the expression of LC3 (Figures 7D,E).

## Discussion

As an important pathogenic process in Parkinson’s disease, neuroinflammation has received increasing attention (De Virgilio et al., 2016). Excessive and persistent inflammation often accelerates the progression of PD pathology and is the main factor of Parkinson’s disease. In addition to neuroinflammation, Parkinson’s disease also involves a variety of pathological reactions, including the accumulation of α-syn protein, mitochondrial dysfunction, calcium disorders and autophagy levels (Lv et al., 2020), that ultimately promote the occurrence of further chronic inflammation. Autophagy is a systematic conservative process that controls the degradation of subcellular components and is involved in the pathological mechanisms of a variety of neurodegenerative diseases, such as Parkinson’s disease, Alzheimer’s disease, Huntington’s disease, and so on (Mizushima and Komatsu, 2011; Martin et al., 2015; Li et al., 2017; Cerri and Blandini, 2019). Previous studies have shown that autophagy is involved in the regulation of a variety of pathological responses of Parkinson’s disease and is mainly involved in the regulation of inflammation (Han et al., 2019; Yao et al., 2019). Autophagy can directly degrade α-syn, the toxic aggregating protein in PD, and delay the activation of microglia (Su et al., 2016; Moors et al., 2017). The elimination of abnormal and damaged mitochondria by selective autophagy (mitochondrial phagocytosis) is not only the main mechanism of mitochondrial quality control but also a pathological manifestation of the progression of PD (Luoma et al., 2004). In PD, imbalances in calcium homeostasis lead to the initiation of autophagy and the activation of inflammatory bodies (Gómez-Suaga and Hilfiker, 2012). In addition, many studies have shown that common abnormal mutations in PD-linked genes, such as SNCA (alpha-synuclein), can lead to the imbalance of autophagy and accelerate the progression of disease, which should be the reason for the decrease in basic autophagic activity that has been observed in patients with PD (Senkevich and Gan-Or, 2020). At the same time, mutations in genes such as SNCA can also increase oxidative stress and neurodegeneration in PD (Chang and Chen, 2020), which also shows that there is a mutual regulatory relationship between autophagy and inflammation.

Earlier studies have shown the relationship between TREM2 and inflammation. TREM2 is an anti-inflammatory protein. For example, TREM2 inhibits the proinflammatory response through PI3K/NF-κB signal transduction (Bonifati et al., 2003). TREM2 inhibits LPS-induced neuroinflammation through the TLR4/NF-κB pathway (Zhang et al., 2019). Calcitonin inhibits LPS-induced proinflammatory cytokines and macrophage activation through TREM2 (Bandow et al., 2020). Moreover, a loss of function mutation of TREM2 leads to the disordered expression of autophagy-related proteins such as LC3, Beclin1, and p62, which indicates that TREM2 is involved in the regulation of autophagy (Satoh et al., 2014).

In our study, we found that autophagy decreased and inflammation increased in LPS-induced BV2 cells and MPTP-treated SNpc cells. Previous studies have shown that TREM2 plays a regulatory role in autophagy in many diseases (Satoh et al., 2014; Ulland et al., 2017). Our research shows that TREM2 is involved in the regulation of autophagy in the progression of PD. The change trend of TREM2 expression in LPS-induced BV2 cells and MPTP-treated mouse SNpc cells tended to be consistent with that of LC3 protein, which suggests that TREM2 is involved in the regulation of autophagy in PD. At the same time, the expression of TREM2 in BV2 cells treated with LPS decreased, while the expression of the inflammatory cytokines TNF-α and IL-1B increased. Our study demonstrated that TREM2 plays a role in regulating inflammation in PD.

To further verify how TREM2 plays the above role, we performed the following studies: we increased the level of TREM2 in BV2 cells by transfection with a TREM2 overexpression plasmid and promoted the overexpression of TREM2 in the mouse midbrain by lentiviral transfection. It should be noted that according to our experimental results, lentivirus infects cells in the mouse brain, including microglia and neurons, but under normal conditions, TREM2 is only expressed in microglia (Casali and Reed-Geaghan, 2021; Diaz-Lucena et al., 2021; Lv et al., 2021). This makes it impossible to prove that *in vitro* data is paralleled by the *in vivo* data. Neurons overexpressed TREM2 may have affected the data of *in vivo* experiments. Previous experiments have also shown that LPS treatment can significantly inhibit autophagy in BV2 cells, while MPTP can significantly inhibit autophagy in frontal cells in the substantia nigra of the midbrain, which seems to be related to the activation of microglia in the substantia nigra (Yao et al., 2019). We constructed a PD model based on LPS and MPTP stimulation. The results of both the *in vivo* and *in vitro* experiments showed that, compared with that of the control group, the ratio of LC3-II/I was increased when TREM2 was highly expressed, which indicates that TREM2 increases the level of autophagy of microglia in an inflammatory environment. Earlier studies have shown that autophagy is mainly regulated by the classic mTOR autophagy pathway (Munson and Ganley, 2015), and our results also prove that the enhancement of autophagy by TREM2 is mediated through the mTOR pathway. Furthermore, we also found that TREM2 overexpression inhibited the decrease in the expression of the inflammatory factors TNF-α and IL-1β, which indicates that the activation of microglia induced by LPS was inhibited by TREM2. In addition, in the MPTP-treated mouse PD model, mesencephalic microglia were significantly activated (Gu et al., 2021), but MPTP-induced microglial activation was inhibited after transfection with TREM2 lentivirus, which is consistent with the results of our *in vitro* experiments. After this experiment, we found that the overexpression of TREM2 not only inhibits the mTOR pathway but also inhibits the activation of the p38 MAPK pathway through decreases p-p38 level. There is a synergistic effect between the p38 MAPK pathway and mTOR pathway in many diseases (Wang et al., 2011; Li et al., 2015), and we found the synergistic effect is involved in the activation of microglia. Overexpression of TREM2 can break this reaction and inhibit the pathological reaction of PD.

Based on our results, we assessed the potential use of TREM2 as an anti-inflammatory and neuroprotective agent. In the MPTP-induced PD model, the apoptosis of dopaminergic neurons after the overexpression of TREM2 was significantly lower than that in the control group. This suggests that in the MPTP model, TREM2 can prevent DA neurons death and apoptosis after the activation of microglia, which could be due to the inhibition of the release of inflammatory cytokines by TREM2. Furthermore, the motor function of PD model mice overexpressing TREM2 was improved compared with that of the control group.

In conclusion, we determined the correlation between TREM2 and microglial activation in the progression of PD and demonstrated that the expression of TREM2 can inhibit the activation of microglia by inhibiting the p38 MAPK pathway; on the other hand, TREM2 can reduce the damage caused by the pathological processes of PD by inhibiting autophagy in the microglia, which is induced by inhibition of the mTOR pathway, and synergistically inhibiting the activation of microglia in the progression of PD. Protect dopaminergic neurons and improve motor symptoms in mice. In summary, TREM2 can affect microglial autophagy and activation through the p38 MAPK/mTOR pathway, thus affecting the pathological changes associated with PD, and this process is likely to be an important link in the inflammatory mechanism of PD.

## Data Availability Statement

The original contributions presented in the study are included in the article/supplementary material, further inquiries can be directed to the corresponding author.

## Ethics Statement

The animal study was reviewed and approved by the Ethics Committee of the Second Affiliated Hospital of Nanchang University.

## Author Contributions

GL, WH, and QL conceived and designed the experiments. QL, YX, ZZ, BH, and SY performed the experiments. YY, JZ, TS, and LJ provided experimental technical support and assisted in completing the study at different stages. QL wrote the manuscript. GL and WH made revisions to the manuscript. All authors read and approved the final manuscript.

## Conflict of Interest

The authors declare that the research was conducted in the absence of any commercial or financial relationships that could be construed as a potential conflict of interest.

## Publisher’s Note

All claims expressed in this article are solely those of the authors and do not necessarily represent those of their affiliated organizations, or those of the publisher, the editors and the reviewers. Any product that may be evaluated in this article, or claim that may be made by its manufacturer, is not guaranteed or endorsed by the publisher.
